# Effects of Individualized Low-Intensity Exercise and Its Duration on Recovery Ability in Adults

**DOI:** 10.3390/healthcare9030249

**Published:** 2021-03-01

**Authors:** Doowon Lee, Ju-Yeon Son, Hyo-Myeong Ju, Ji-Hee Won, Seung-Bo Park, Woo-Hwi Yang

**Affiliations:** Graduate School of Sports Medicine, CHA University, Seongnam-si 13503, Gyeonggi-do, Korea; doowonlee221@chauniv.ac.kr (D.L.); juyeonson@chauniv.ac.kr (J.-Y.S.); pagooo@chauniv.ac.kr (H.-M.J.); jiheewon7@chauniv.ac.kr (J.-H.W.); parks0524@naver.com (S.-B.P.)

**Keywords:** aerobic exercise, endurance, fat oxidation, lactate threshold, regenerative endurance

## Abstract

Exercise is recommended to increase physical health and performance. However, it is unclear how low-intensity exercise (LIE) of different durations may affect or improve recovery ability. This study aimed to investigate how LIE-duration with the same volume affects recovery ability in adults. Twenty healthy male adults participated in this study. Participants were randomly assigned to the 30-min (*n* = 10) or the 1-h LIE group (*n* = 10). The intervention included sixteen exercise sessions/four weeks with a 30-min LIE group, and eight exercise sessions/four weeks with a 1-h LIE group. Heart rate (HR) corresponding to <2 mmol∙L^−1^ blood lactate (La^−^) was controlled for LIE. Pre- and post-testing was conducted before and after 4-week LIE and tests included jogging/running speed (S), HR, and differences (delta; ∆) in HR and S between pre- and post-testing at 1.5, 2.0, and 4.0 mmol∙L^−1^ La^−^. Only the HR at 2.0 mmol∙L^−1^ La^−^ of the 30-min LIE group was decreased in the post-test compared to the pre-test (*p* = 0.043). The jogging/running speed of the 1-h LIE group was improved in the post-test compared to the pre-test (*p* < 0.001, *p* = 0.006, *p* = 0.002, respectively). ∆HR at 2.0 and ∆S between the 30-min and 1-h LIE group at 1.5, 2.0, and 4.0 mmol∙L^−1^ La^−^ were significantly different (*p* = 0.023, *p* < 0.001, *p* = 0.002, and *p* = 0.019, respectively). Furthermore, moderate to high positive correlations between ∆HR and ∆S of all subjects at 1.5 (*r* = 0.77), 2.0 (*r* = 0.77), and 4.0 (*r* = 0.64) mmol∙L^−1^ La^−^ were observed. The 1-h LIE group showed improved endurance not only in the low-intensity exercise domain, but also in the beginning of the moderate to high-intensity exercise domain while the 30-min LIE group was not affected by the 4-week LIE intervention. Therefore, LIE (<2.0 mmol∙L^−1^) for at least 1-h, twice a week, for 4 weeks is suggested to improve recovery ability in adults.

## 1. Introduction

The goal of prescribed exercise is to improve physical health and performance in the general population and athletes [1,2]. Sports scientists and clinical physicians have used lactate threshold (LT) tests for over fifty years due to their application as a useful metric for recommendations on individualized exercise intensity in elite athletes and cardiac patients [3,4]. A rightward shift of the exponential lactate curve is generally interpreted as improved endurance performance [5,6,7,8,9]. Furthermore, the blood lactate level during exercise is a more sensitive biomarker of endurance performance than maximal oxygen uptake (VO_2max_) [10]. Indeed, a recent study reports that non-exercise based maximal heart rate (HR_max_) and VO_2max_ equations are not suitable for individualized exercise prescription in the clinical setting [11]. 

Validated LT concepts such as anaerobic, aerobic–anaerobic threshold, maximal lactate steady state (MLSS), and the onset of blood lactate accumulation (OBLA; 4 mmol·L^−1^) were developed and refined by several groups [5,12,13,14,15,16]. However, some of these concepts have been controversial [9,14]. Despite several generally used definitions of the aerobic–anaerobic transition, this biochemical phenomenon does not actually exist because the glycolytic pathway is accelerated to supply adenosine triphosphate (ATP) for active skeletal muscles during increments in intense exercise [9,14,17]. 

Nowadays, the blood lactate level of the LT test is used to differentiate exercise intensity areas, which include low-intensity (<2 mmol·L^−1^; zone 1; LIE), moderate-intensity (2–4 mmol·L^−1^; zone 2; MIE), and high-intensity exercise (>4 mmol·L^−1^; zone 3; HIE) [9,15,18,19,20]. HIE induces dramatic effects on maximal aerobic performance, which is achievable through relatively short exercise times (volume). However, HIE may be unsuitable for exercise beginners and/or the general population with relatively poor endurance performance because it causes faster depletion of carbohydrates, stress on skeletal muscle, high risk of muscle injury, and dysbalance of hormones [21,22,23]. During MIE, carbohydrates and fat are used as the main energy sources and represent the predominant energy source under aerobic conditions [9,24,25]. Therefore, high exercise volumes at MIE are difficult for exercise beginners and the general population [9,24,25]. Previous studies have shown that threshold training produces the lowest effects of exercise compared to HIE, high volume training (HVT), and polarized training (POT) in athletes and obese women [19,26]. 

In comparison to HIE and MIE, fat oxidation is the predominant energy source used in LIE (<2 mmol·L^−1^) and LIE is commonly interpreted as recovery exercise zone [4,9]. In this regard, triglycerides in adipocytes are hydrolyzed into glycerol and free fatty acids (FFAs) that are converted into acetyl-CoA by beta-oxidation in the mitochondria [9,27,28]. In contrast, blood lactate values start to decrease in LIE as more pyruvate and lactate are utilized by the aerobic pathway than in anaerobic glycolysis [29]. However, the predominant activation of these mechanisms during exercise also seems to be dependent on exercise duration [9,24,30]. van Loon et al. [30] suggested that fat oxidation provided 66% of total energy demand at rest. Furthermore, FFAs and intracellular lipid during MIE contributed 48% and 17%, respectively. Other outcomes of previous studies have shown that LIE (40% of VO_2max_ and 50%–60% of maximal heart rate) needs to be performed 4–6 h per week over 12 weeks in obese men, which improved fat metabolism [31,32]. This increased fat metabolism plays a crucial role in the physiological mechanisms to improve recovery ability because fat oxidation supports the Cori cycle in the liver and kidney (cell-to-cell lactate shuttle mechanism; re-synthesis) and influences the activation of key enzymes and hormonal responses of gluconeogenesis [9,27,33,34,35,36]. Hence, reduced blood lactate should be an indicator of the efficient use of fat oxidation and enhanced recovery during LIE. In particular, both elite athletes and the general population need to perform LIE in order to improve regenerative endurance, which consists of the ability to effectively perform fat oxidation and ATP re-synthesis [4,9].

However, based on the aforementioned scientific outcomes, it is unclear as to how the duration and frequency (volume) of individualized LIE (<2 mmol·L^−1^) impacts recovery ability in the general population because there are relatively fewer studies regarding LIE. Furthermore, previous studies have conducted different low-intensity exercises based on the percentages of estimated HR_max_ and VO_2max_. Therefore, the aim of this study was to investigate, using the LT test, how different exercise duration (30 min vs. 1 h) with the same exercise volume (4 weeks) affects recovery ability in adults.

## 2. Materials and Methods

### 2.1. Subjects

A total of twenty healthy male adults (*n* = 20) participated in this study and they were recruited from regional fitness centers through official research notifications. Subjects were randomized to separate 30-min (*n* = 10) and 1-h LIE groups (*n* = 10). The anthropometric parameters of the subjects were as follows (mean ± SD): 30-min LIE group, age: 28.3 ± 2.0 years, height: 173.5 ± 4.4 cm, weight: 75.9 ± 9.9 kg, fat: 16.3 ± 3.9%, BMI: 25.1 ± 2.2 kg∙m^2^; 1-h LIE group, age: 28.8 ± 6.9 years, height: 173.4 ± 3.9 cm, weight: 75.4 ± 8.9 kg, fat: 17.7 ± 5.9%, BMI: 25.0 ± 2.3 kg∙m^2^ (pre- and post-intervention data in Table 1). All were had been involved in strength training for at least six months and the training volume was between 16–18 h per week. Subjects did not take any medication during all procedures and abstained from alcohol and nicotine consumption for four weeks of exercise intervention and at least 24 h before the experiment. Furthermore, subjects did not consume any liquid during all tests. The study was approved by the Institutional Ethics Committee of the CHA University (No. 1044308-202003-HR-007-02). The applied protocols align with the Declaration of Helsinki.

### 2.2. Study Design

**Figure 1:**
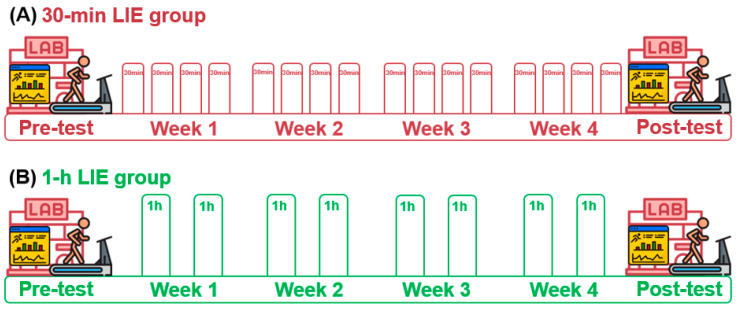
Study design (**A**) 30-min group: 4 sessions × 4 weeks of low-intensity exercise (<2 mmol∙L^−1^) (**B**) 1-h group: 2 sessions × 4 weeks of low-intensity exercise (<2 mmol∙L^−1^). The pre-test was conducted before the 4-week exercise intervention. After the 4-week exercise intervention, all subjects performed the post-test. LIE: low-intensity exercise.

In the study, two measurement points were scheduled for the 30-min and 1-h LIE groups (pre- and post-test; Figure 1). All procedures were conducted and controlled in the same laboratory environment throughout the experiment (temperature: 23 °C; relative humidity: 50%). All subjects were instructed not to alter their diet throughout the exercise phase and to maintain strength training if it was part of their usual exercise program. The nutritional intake of subjects was not controlled during the study, however, food intake was not allowed for a three-hour period before all testing [37]. Subjects completed an anthropometric measurement using a bioelectrical impedance analysis (Inbody 270; Inbody Co. Ltd., Seoul, Korea), and the LT test [12,16] was performed on a treadmill (NR30XA, DRAX Corporation Ltd., Seoul, Korea). As an exercise control, HR assessment was monitored during each LIE session, and the results (exercise mod, duration, and intensity) were digitally saved in the associated HR application [37].

### 2.3. Intervention of Low-Intensity Exercise 

To achieve the same exercise volume in the different intervention groups, the four weeks consisted of sixteen exercise sessions of 30-min LIE or eight exercise sessions of 1-h LIE. Four-week exercise interventions were selected based on previous studies and in order to study the effects of maintaining the same exercise volume [2,19,37,38]. Exercise intensity (LIE with heart rate (HR) corresponding to individualized <2 mmol∙L^−1^ blood lactate) was controlled by HR based on the LT test at the pre-test [18,19,26,37,39]. Percentages of estimated maximal heart rate were calculated [40] (Table 2).

### 2.4. Laboratory Pre- and Post-Test 

The pre-test subjects performed an incremental exercise on a treadmill (LT test), which consisted of 5-min stages with 30 s breaks between stages. The first stage was started at 1.0 m∙s^−1^, with increments of 0.5 m∙s^−1^ every 5 min. The criteria for ending testing were a blood lactate concentration over 4 mmol∙L^−1^ after each running speed or until volitional exhaustion [12,16,37]. After four weeks of LIE, the same set-up was used for the post-test. HR and jogging/running speed at the 1.5, 2.0, and 4.0 mmol∙L^−1^ blood lactate concentration levels (La^−^) were estimated using a mathematical model of the interpolation that has previously been explained in detail [16,41,42,43]. Delta (∆) jogging/running speed and ∆HR at the 1.5, 2.0, and 4.0 mmol∙L^−1^ La^−^ between pre- and post-testing were calculated (S_1.5_, S_2_, S_4_, ∆S_1.5_, ∆S_2_, ∆S_4_ and HR_1.5_, HR_2_, HR_4_, ∆HR_1.5_, ∆HR_2_, ∆HR_4_). The HR of all subjects was recorded using HR telemetry (H10 sensor, Polar Electro, Finland). The mean value of HR over the last 30 s of each stage was determined for statistical analysis. Capillary blood sampling for lactate analysis was taken from the earlobe (0.2 µL) immediately after each 5-min stage. All blood lactate levels were determined by an enzymatic-amperometric method (Lactate Scout 4, EKF-diagnostics GmbH, Germany) [44,45].

### 2.5. Statistical Analyses

All data were analyzed using GraphPad Prism 9.0 (GraphPad Software, La Jolla, CA, USA). Parameters are presented as mean and standard deviation (SD)/standard error of the mean (S.E.M). Normal distribution was performed using the Shapiro–Wilk test. A paired *t*-test was used to compare the pre-test and post-test within each group. Furthermore, an independent *t*-test was utilized to compare the differences between the 30-min LIE and 1-h LIE groups. Box and whisker plots indicate minimum to maximum and median values. Effect sizes (ES; Cohen’s *d*) were calculated for parametric tests and thresholds for small, moderate, and large effects were 0.2, 0.5, and 0.8, respectively [46]. Differences were considered significant at *p* < 0.05 and *p* < 0.01. Additionally, a Pearson’s two-tailed correlation was performed between ∆S and ∆HR at 1.5, 2.0, and 4.0 mmol∙L^−1^ La^−^. 

## 3. Results

### 3.1. Comparison of Pre- and Post-Test HR and Jogging/Running Speed of 30-min LIE Group

**Figure 2:**
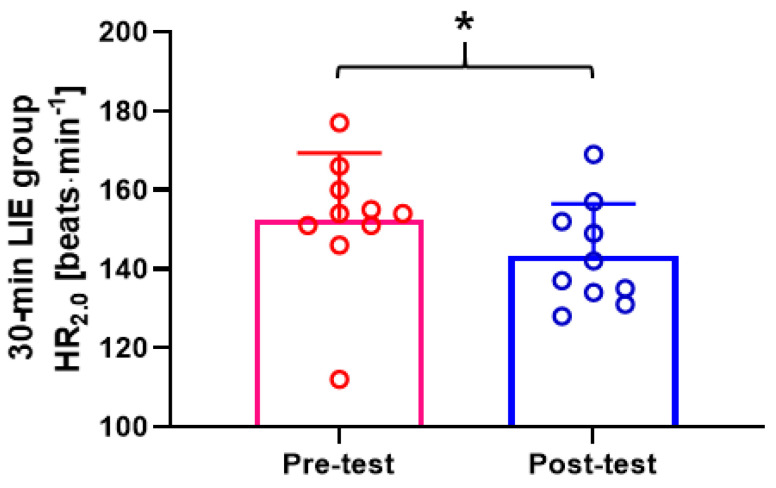
Heart rate (HR) at 2.0 mmol∙L^−1^ La^−^ of 30-min LIE group. During the post-test, HR at 2.0 mmol∙L^−1^ La^−^ was decreased in comparison to the pre-test (*p* = 0.043). Data are mean ± SD. * *p* < 0.05.

HR at 2.0 mmol∙L^−1^ La^−^ of the 30-min LIE group was decreased in the post-test compared to the pre-test (*p* = 0.043; ES: 0.66) (Figure 2). Other HR data at 1.5 and 4.0 mmol∙L^−1^ La^−^ were not affected by the 30-min LIE intervention (*p* > 0.05). Furthermore, the jogging/running speed at 1.5, 2.0, and 4.0 mmol∙L^−1^ La^−^ showed no significant change between pre- and post-testing (*p* > 0.05). 

### 3.2. Comparison of Pre- and Post-Test HR and Jogging/Running Speed of 1-h LIE Group

**Figure 3:**
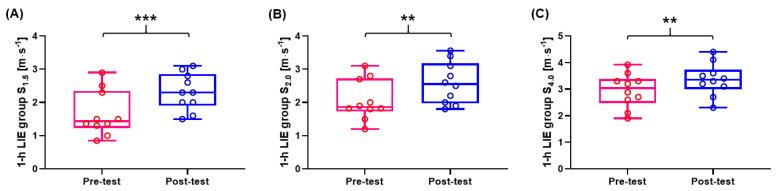
Jogging/running speed data of 1-h LIE group at 1.5, 2.0, and 4.0 mmol∙L^−1^ La^−^. Jogging/running speed was significantly improved at (**A**) 1.5 mmol∙L^−1^ La^−^ (*p* < 0.001) (**B**) 2.0 mmol∙L^−1^ La^−^ (*p* = 0.006) and (**C**) 4.0 mmol∙L^−1^ La^−^ (*p* = 0.002). ** *p* < 0.01 and *** *p* < 0.001.

**Figure 4:**
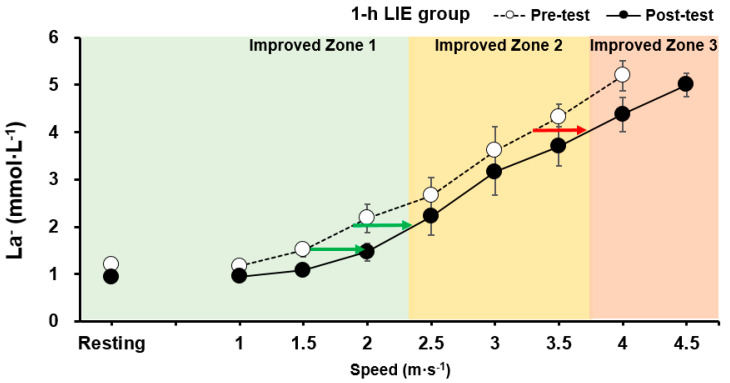
Improved jogging/running speed of 1-h LIE group at 1.5, 2.0, and 4.0 mmol∙L^−1^ La^−^ was associated with an enhanced rightward shift of the exponential lactate curve and exercise intensity zone. Data are mean ± S.E.M.

There was no significant difference in HR at 1.5, 2.0, and 4.0 mmol∙L^−1^ La^−^ between the pre- and post-test (*p* > 0.05). However, the jogging/running speed of the 1-h LIE group at 1.5, 2.0, and 4.0 mmol∙L^−1^ La^−^ was significantly increased post-intervention (*p* < 0.001; ES: 1.05, *p* = 0.006; ES: 0.86, *p* = 0.002; ES: 0.65, respectively). Data are presented in Figure 3A–C and Figure 4. 

### 3.3. Comparison of HR, Jogging/Running Speed, ∆HR, and ∆Jogging/Running Speed between 30-min and 1-h LIE Group

**Figure 5:**
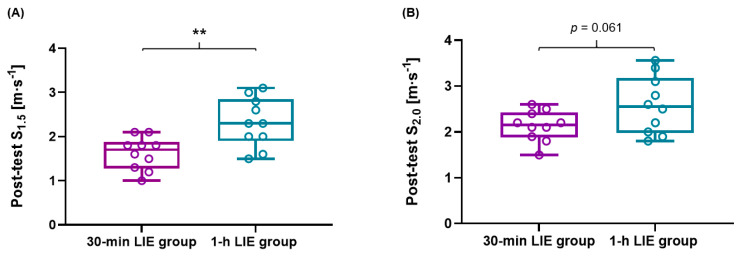
Jogging speed at 1.5 and 2.0 mmol∙L^−1^ La^−^ between 30-min LIE and 1-h LIE groups. (**A**) In the post-test of the 1-h LIE group, jogging speed at 1.5 mmol∙L^−1^ La^−^ was significantly increased compared to the 30-min LIE group (*p* = 0.004). (**B**) Jogging speed at 2.0 mmol∙L^−1^ La^−^ tended to increase in the 1-h LIE group compared to the 30-min LIE group (*p* = 0.061). ** *p* < 0.01.

Jogging/running speed and HR changes post-intervention at 2.0 and 4.0 mmol∙L^−1^ La^−^ showed no differences between short and long exercise duration (*p* > 0.05). However, jogging speed at 1.5 mmol∙L^−1^ La^−^ of the 1-h LIE group was significantly higher compared to the 30-min LIE group (*p* = 0.004; ES: 1.47) (Figure 5A). Delta HR at 2.0 mmol∙L^−1^ La^−^ and delta jogging/running speed at 1.5, 2.0, and 4.0 mmol∙L^−1^ La^−^ of the 1-h LIE were significantly higher compared to the 30-min LIE group (*p* = 0.023; ES: 1.11, *p* < 0.001; ES: 1.85, *p* = 0.002; ES: 1.58, *p* = 0.019; ES: 1.16, respectively) (Table 3).

### 3.4. Correlations between ∆HR and ∆S of All Subjects at 1.5, 2.0, and 4.0 mmol·L^−1^ La^−^

**Figure 6:**
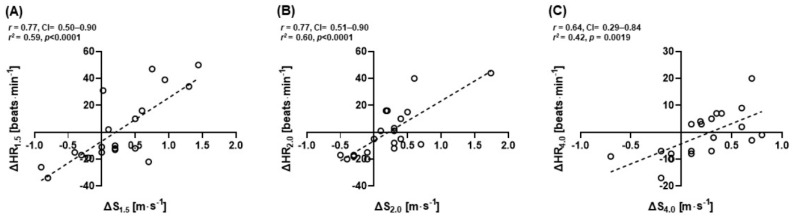
Pearson’s two-tailed correlation (*n* = 20) between delta (∆) jogging/running speeds and delta (∆) heart rate at (**A**) 1.5 (*r* = 0.77, *p* < 0.0001), (**B**) 2.0 (*r* = 0.77, *p* < 0.0001), and (**C**) 4.0 mmol∙L^−1^ La^−^ (*r* = 0.64, *p* = 0.0019).

Moderate to high positive correlations between ∆HR and ∆S of all subjects at 1.5 (*r* = 0.77; 95% CI: 0.50–0.90; *r*^2^ = 0.59; *p* < 0.0001), 2.0 (*r* = 0.77; 95% CI: 0.51–0.90; *r*^2^ = 0.60; *p* < 0.0001), and 4.0 mmol∙L^−1^ La^−^ (*r* = 0.64; 95% CI: 0.29–0.84; *r*^2^ = 0.42; *p* = 0.0019) were observed (Figure 6A–C).

## 4. Discussion

The effect of LIE-duration and frequency on recovery ability is currently controversial. Therefore, the present study examined how a different LIE-duration with the same exercise volume (4 × 4 weeks; 30-min LIE group and 2 × 4 weeks; 1-h LIE group) influences the recovery ability in male adults. The major findings show that jogging/running speed at 1.5, 2.0, and 4.0 mmol∙L^−1^ La^−^ of the 1-h LIE group was improved after the 4-week exercise intervention. Also, values of ∆HR_2_, ∆S_1.5_, ∆S_2_, and ∆S_4_ in the 1-h LIE group were significantly higher compared to the 30-min LIE group.

HR values showed only a decreased HR_2.0_ and ∆HR_2.0_ in the 30-min LIE group and between both groups, while no significant difference in jogging speed at 2 mmol∙L^−1^ La^−^ was observed. HR levels of the 1-h LIE group were tended to increase with improved jogging/running speeds in the post-test, but no significant change was found. Indeed, our correlation analyses showed positive, moderate to high relationships between ∆HR and ∆S at certain lactate levels. (Figure 3 and Figure 6, and Table 3). Increased and decreased HR levels after 4-week LIE intervention may be caused by altered cardiac sympathetic and parasympathetic modulation [47,48,49]. However, these outcomes (tendency) should be considered with other HR-related parameters such as heart rate variability (HRV), blood pressure, and HR_max_ in future studies. Accordingly, most earlier studies regarding LIE have been conducted in cardiac patients in which several parameters such as HR_max_, HRV, and blood pressure were analyzed [50,51,52]. HRV was positively affected after five sessions of LIE per week for 12 weeks in patients with peripheral artery disease, which increased the parasympathetic activation [51]. Furthermore, patients with chronic heart failure and hypertension performed 3–5 sessions of 1-h LIE for 12 weeks (40% of VO_2max_ and 40% of HR_max_, respectively), which resulted in increased left ventricular ejection, decreased blood pressure, and HRV interval [50,52]. In this regard, LIE seems to influence the HR variables with a higher exercise volume of at least 4–5 sessions per week over 12 weeks. 

Jogging/running speed at 1.5, 2.0, and 4.0 mmol∙L^−1^ La^−^ of the 30-min LIE group was not affected by the 4-week LIE intervention. In contrast, jogging/running speed at 1.5, 2.0, and 4.0 mmol∙L^−1^ La^−^ of the 1-h LIE group in the post-test was significantly enhanced (see Figure 3). The recommended HR of the 1-h LIE group was 62% of HR_max_ in our study (Table 2). Similarly, a previous study has reported that 40-min LIE (64% of HR_max_) for four weeks in cardiac patients could increase the running speed corresponding to 4 mmol∙L^−1^ La^−^ by 5% [2]. Furthermore, LIE in recreational runners (60%–70% of HR_max_; 30–60 min; eight weeks) caused a reduction in accumulated blood lactate and rate of blood lactate production during the graded exercise test by 70% and 57%, respectively [53]. Another study has shown that POT that included over 80% LIE (<2 mmol·L^−1^; zone 1) after three weeks resulted in improved time to exhaustion by 5% and increased jogging speed at 2.0 mmol∙L^−1^ La^−^ by 9% [37]. Hommel et al. [39] showed that healthy male adults increased the power in MLSS after 1-h LIE at 1.5 to 2.5 mmol∙L^−1^ La^−^, three days per week for 4 and 6 weeks. To improve endurance performance, the distribution of training intensity in world-class middle- and long-distance runners suggests over 87% of zone 1 (<2 mmol·L^−1^) per week from the general preparation phase [54]. 

Previous studies suggest that total fat utilization is increased while carbohydrate usage is decreased by endurance training [24,55,56,57]. Thus, it is understandable why the jogging/running speed of the 1-h LIE group at 1.5, 2.0, and 4.0 mmol∙L^−1^ La^−^ increased in the present study, which also indicates an improved rightward shift of the exponential lactate curve (Figure 4) [5,6,7,8,9,55,56,57]. In contrast, the jogging/running speed of the 30-min LIE group was not statistically different between pre- and post-testing. In light of this, LIE of 25% VO_2max_ mainly induces delivery of plasma fatty acids for energy production, while the production of lactate is decreased for ATP re-synthesis [9,27,28]. 

Under resting and LIE conditions, fat and lactate metabolism is largely dependent on mitochondrial abundance and function [9,58]. During LIE, the resting lactate is predominantly transported via blood from muscle cells to the liver/kidney (Cori cycle) transport, which is supported by the increased hepatic blood flow. In contrast, lactate from muscle cells is resynthesized less by the intracellular lactate shuttle mechanism during LIE [9,59,60]. Furthermore, key enzymes and hormonal responses of gluconeogenesis such as pyruvate kinase, pyruvate carboxylase, phosphoenolpyruvate carboxykinase, glucagon, cortisol, and other associated regulators such as cyclic adenosine monophosphate and intracellular calcium are activated by FFA [61,62,63,64,65]. In contrast, FFA inhibits glycolysis-related enzymes such as pyruvate dehydrogenase [9,61,62,63,64,65]. 

San-Millan et al. [66] and Yang et al. [9] have suggested that the general population has relatively poor recovery ability (lower fat oxidation and re-synthesis from lactate) compared to professional athletes during LIE. Also, these outcomes result in indirect aerobic conditions in different populations, which are associated with the aforementioned mechanisms in mitochondria. The high negative correlation between blood lactate and fat oxidation (*r* = −0.92–−0.98) are seen in different populations such as elite cyclists, moderately active male individuals, and individuals with metabolic syndrome [66]. Thus, the improved jogging/running speed at a different blood lactate concentration in the 1-h LIE group for 4 weeks can be interpreted as due to the increased utilization of fat and recovery ability in male adults. Maximal fat oxidation can be reached with a prolonged exercise duration of over 1-h at moderate intensity (65% of VO_2max_) [67]. Furthermore, the outcomes of van Loon et al. [30] showed that the highest total fat oxidation (FFA and intramyocellular lipids) in male cyclists during MIE occurred after 120 min. On the other hand, muscle glycogen decreased with increased duration. These results indicate that the absolute use of fat oxidation during LIE is relatively lower than during MIE. However, fat oxidation can predominantly be used during LIE because of the very low utilization of carbohydrates as an energy source and unnecessary carbohydrates can be efficiently saved [24,25]. Hence, LIE requires a longer exercise duration to achieve the efficient use of fat oxidation compared to MIE [9,24,25]. However, LIE of only 30-min duration for four weeks does not sufficiently improve metabolic flexibility, which reflects the ability to oxidize fat and carbohydrate, mitochondrial function, and oxidative capacity including fat oxidation. This is seen in our data (Figure 5 and Table 3) and also confirmed in previous studies [24,30].

Our findings indicate that 1-h LIE can improve fat oxidation and ATP re-synthesis, resulting in enhanced recovery ability and general aerobic endurance in adults. Further studies are expected to investigate how these metabolic changes are associated with 1-h LIE using other metabolic-related parameters such as respiratory exchange ratio and blood glucose.

## 5. Conclusions

The findings of the present study indicate that the 1-h LIE group improved endurance, not only in the low-intensity exercise domain but also in the beginning of the moderate to high-intensity exercise zones (zones 1, 2, and 3). Thus, LIE (<2.0 mmol∙L^−1^) for at least 1-h, twice a week, for 4 weeks is suggested to enhance recovery ability and endurance performance in adults. As well, LIE may be suitable not only for athletes but also pregnant women, exercise beginners, older adults and even cardiac patients, because of the very low propensity for muscle injury and cardiometabolic risk. Further studies are expected to investigate how the volume (duration and frequency) of LIE influences other specific populations such as older adults and cardiac patients.

## Figures and Tables

**Table 1:** Anthropometric data at pre- and post-testing of 30-min low-intensity exercise (LIE) (*n* = 10) and 1-h LIE (*n* = 10) groups.

Parameters	30-min LIE Group (Mean ± SD)		1-h LIE Group (Mean ± SD)	
	Pre-Test	Post-Test	Pre-Test	Post-Test
Age (years)	28.3 ± 2.0		28.8 ± 6.9	
Height (cm)	173.5 ± 4.4		173.4 ± 3.9	
Body weight (kg)	75.9 ± 9.9	76.1 ± 11.2	75.4 ± 8.9	75.7 ± 9.56
Body fat (%)	16.3 ± 3.9	16.4 ± 4.3	17.7 ± 5.9	17.8 ± 6.3
BMI (kg∙m^2^)	25.1 ± 2.2	25.2 ± 2.6	25.0 ± 2.3	25.1 ± 2.5

BMI: Body mass index. Anthropometric data was not significantly changed between pre- and post-testing of all groups.

**Table 2:** Recommended heart rate, estimated maximal heart rate percentage, and corresponding jogging speed for low-intensity exercise (<2.0 mmol∙L^−1^).

Group	HR (Beats∙min^−1^)	% of HR_max_	Jogging Speed (m∙s^−1^)
30-min LIE group (*n* = 10)	117 ± 10	66.6 ± 8.71	1.50 ± 0.31
1-h LIE group (*n* = 10)	127 ± 16	62.2 ± 5.75	1.53 ± 0.14

HR: heart rate, LIE: low-intensity exercise, % of HR_max_: estimated maximal heart rate percentages.

**Table 3:** Delta HR and delta jogging/running speed at 1.5, 2.0, and 4.0 mmol∙L^−1^ La^−^ of 30-min LIE (*n* = 10) and 1-h LIE (*n* = 10) groups.

Parameters	30-min LIE Group	% of 30-min G	1-h LIE Group	% of 1-h G	Significance	Effect Size
	Mean ± SD	Mean ± SD	Mean ± SD	Mean ± SD	*p*	*d*
∆HR_1.5_ (beats∙min^−1^)	−10.00 ± 19.35	−5.20 ± 17.56	12.30 ± 27.72	14.99 ± 27.21	0.052	0.93
∆HR_2.0_ (beats∙min^−1^)	−9.20 ± 12.34	−5.45 ± 8.79	9.20 ± 19.95	8.50 ± 16.58	0.023 *	1.11
∆HR_4.0_ (beats∙min^−1^)	−2.70 ± 7.31	−1.38 ± 4.18	0.80 ± 9.65	0.65 ± 5.80	0.37	0.41
∆S_1.5_ (m∙s^−1^)	−0.17 ± 0.45	−6.76 ± 24.04	0.66 ± 0.45	51.66 ± 44.01	<0.001 ***	1.85
∆S_2.0_ (m∙s^−1^)	−0.09 ± 0.28	−2.35 ± 14.20	0.52 ± 0.46	29.03 ± 27.32	0.002 **	1.58
∆S_4.0_ (m∙s^−1^)	0.03 ± 0.34	−1.93 ± 10.73	0.41 ± 0.29	15.31 ± 11.36	0.019 *	1.16

∆HR_1.5_: the difference in heart rate between pre- and post-test at 1.5 mmol∙L^−1^ La^−^, ∆HR_2.0_: the difference in heart rate between pre- and post-test at 2.0 mmol∙L^−1^ La^−^, ∆HR_4.0_: the difference in heart rate between pre- and post-test at 4.0 mmol∙L^−1^ La^−^, ∆S_1.5_: the difference in jogging speed between pre- and post-test at 1.5 mmol∙L^−1^ La^−^, ∆S_2.0_: the difference in jogging speed between pre- and post-test at 2.0 mmol∙L^−1^ La^−^, ∆S_4.0_: the difference in running speed between pre- and post-test at 4.0 mmol∙L^−1^ La^−^, % of 30-min G: percentage changes between pre- and post-test of 30-min LIE group, % of 1-h G: percentage changes between pre- and post-test of 1-h LIE group. * *p* < 0.05, ** *p* < 0.01 and *** *p* < 0.001.

## Data Availability

Data available on request due to restrictions eg privacy or ethical. The data presented in this study are available on request from the corresponding author. The data are not publicly available due to privacy of CHA University.
